# The experience of canakinumab in renal amyloidosis secondary to Familial Mediterranean fever

**DOI:** 10.1186/s40348-016-0058-2

**Published:** 2016-08-15

**Authors:** Betul Sozeri, Nesrin Gulez, Malik Ergin, Erkin Serdaroglu

**Affiliations:** 1 Department of Pediatric Rheumatology, Ege University Faculty of Medicine, Izmir, Turkey; 2 Dr. Behcet Uz Children Diseases Teaching and Research Hospital Pediatric Immunology and Rheumatology, Izmir, Turkey; 3 Dr. Behcet Uz Children Diseases Teaching and Research Hospital Pathology, Izmir, Turkey; 4 Dr. Behcet Uz Children Diseases Teaching and Research Hospital Pediatric Nephrology, Izmir, Turkey

**Keywords:** Familial Mediterranean fever, Amyloidosis, Child, Canakinumab

## Abstract

**Introduction:**

Familial Mediterranean fever (FMF) is an autosomal recessive disease characterized by self-limited recurrent attacks of fever and serositis. Patients may develop renal amyloidosis. Colchicine prevents attacks and renal amyloidosis. Five to 10 % of the patients with FMF are resistant or intolerant to colchicine.

**Case description:**

Herein, we reported our experience with clinical-laboratory features and treatment responses of a pediatric FMF patient with amyloidosis treated with canakinumab. We observed a significant decrease in proteinuria and increase growth in the patient.

**Discussion and evaluation:**

The most serious complication of FMF is the development of AA type amyloidosis which is characterized by proteinuria. Colchicine is the prototype drug that decreases production of amyloidogenic precursor protein. Occasionally, colchicine inadequate patient is observed, as in our case. Canakinumab is a human anti-IL-1β monoclonal antibody. Previously, canakinumab efficacy were shown in a limited number of studies.

**Conclusions:**

Our data, though limited to only one patient, emphasize that therapeutic intervention with canakinumab seems to be improve kidney function in colchicine-resistant FMF with renal amyloidosis.

## Background

Familial Mediterranean fever (FMF) is a genetic, autoinflammatory disease, characterized by acute episodes of serosal and cutaneous inflammation, expressed with pain, fever, neutrophilia, and intense acute-phase response, caused by activation of the innate immune system [1]. The FMF gene, named MEFV, is located on the short arm of chromosome 16 [2, 3]. It encodes a 781–amino acid protein called pyrin or marenostrin which is expressed mostly in neutrophils and acts in controlling inflammation by deactivating inflammatory peptides [4, 5]. Mutated forms of it may be involved in a series of reactions that ultimately enhance the overexpression of IL-1b and consequent inflammation [1].

FMF has been associated with an increased risk for secondary amyloidosis, mainly affecting the renal and vascular function in untreated or insufficiently treated patients with FMF. Amyloidosis is a progressive destructive disorder that results in organ dysfunction due to extracellular deposition of *N* terminal fragments of serum amyloid A protein (SAA) in the form of insoluble amyloid fibrils [6]. Proteinuria is usually the earliest and most common clinical manifestation of AA amyloidosis in patients with inflammatory diseases [6].

The goal of therapy for FMF is the prevention of acute attacks, development, and progression of amyloidosis. Colchicine is the mainstay of therapy which decreases attack frequency and increases the quality of life in more than 60 % of patients [7]. It also prevents SAA secretion and slows the progression of amyloidosis in patients with FMF [7]. Approximately 40 % of FMF patients treated with colchicine have a partial remission showed in the Eurofever study [8]. Also, 5–10 % of the patients were reported to be resistant; suffering from either more than six typical FMF attacks per year or more than three typical FMF attacks within 4–6 months [9]. Several studies showed that the patients with FMF were successfully treated with agents blocking interleukin (IL)-1 activity due to the critical role of IL-1 in the pathogenesis FMF [10–14].

Canakinumab is a high-affinity human anti-IL1 β monoclonal antibody of the IgG1/k isotype developed for the treatment of immune disorders, and it is highly specific for IL-1β and does not interfere with other IL-1-activated pathways.

We would like to share our experience of a patient with colchicine-resistant FMF and renal amyloidosis, whose treatment with canakinumab substantially improved renal functions and reduced proteinuria over a period of 26 months.

## Case presentation

A 14-year-old male with colchicine-resistant FMF and amyloidosis was admitted for the first time in May 2013. He was born to parents of second-degree consanguineous marriage. He had taken regular colchicine therapy (2 g/day) and ramipril (5 mg/day) for proteinuria for a year.

**Fig. 1:**
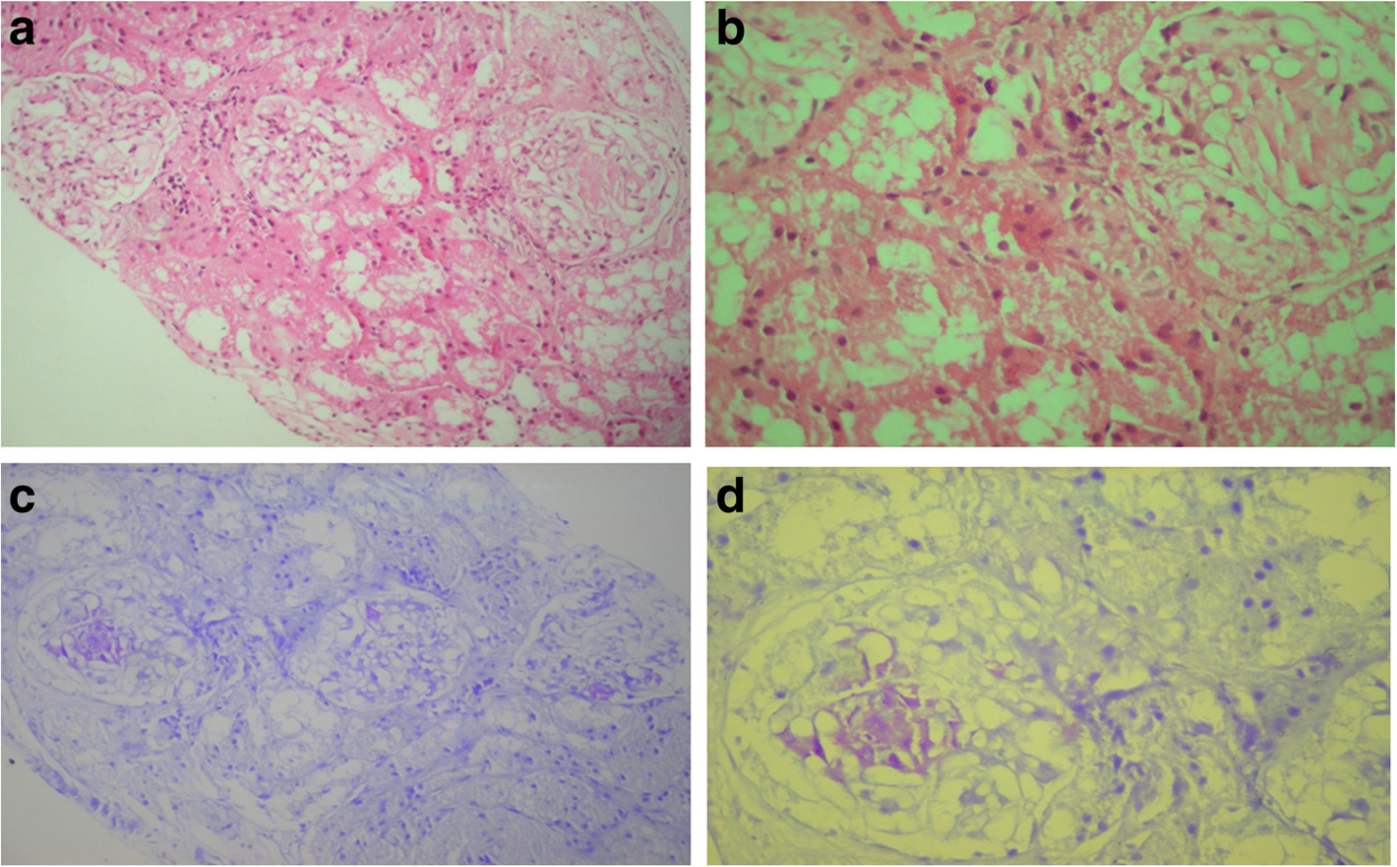
Renal amyloid deposition was diagnosed in glomerulus by two different staining

From his history, he had recurrent FMF attacks associated with severe abdominal pain, joint pain, and fever, which had begun at the age of four. However, he was hospitalized for the first time at the age of 7 years because of intermittent febrile episodes with chills, abdominal pain, and arthritis involving ankle joints. Splenomegaly was found a year later. His attacks continued once a month until he was diagnosed with FMF at 13 years old. At that time, he had proteinuria, splenomegaly, and growth retardation. He was homozygous for the M694V mutation in MEFV gene. Also, microhematuria and proteinuria (38 mg/m2/h) was found at his urinalysis. Serum creatinine level was 1.1 mg/dl, and creatinine clearance was 89 mL/min. Diagnosis of chronic kidney disease (CKD) with AA amyloidosis was established via renal biopsy (Fig. 1). Severe glomerular amyloidosis plus mild vascular and mild interstitial amyloidosis was found in his kidney biopsy. Colchicine therapy was started with a dose of 1 mg/day. In the first month of treatment, he was clinically normal and his C-reactive protein (CRP) level was within normal limits. At his 6-month follow up, the dose of colchicine had to be increased (2 g/day) due to an increased attack rate (5 attacks per 6 months) despite the regular use of drugs.

**Fig. 2:**
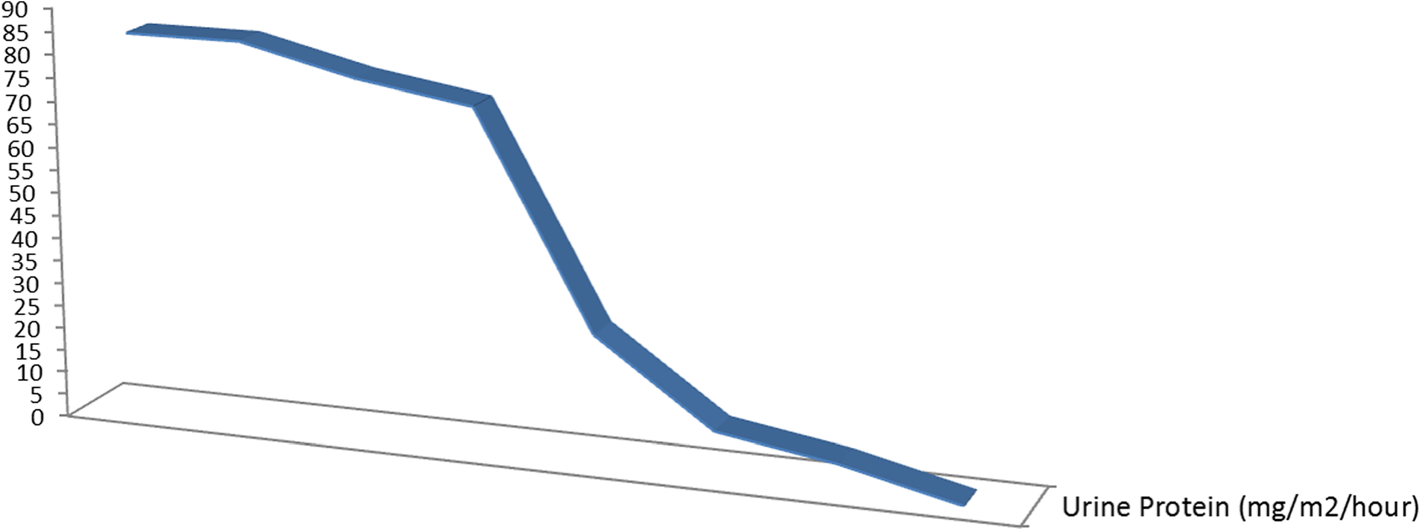
Effect of canakinumab on proteinuria (miligrams/m2(per hour)

**Fig. 3:**
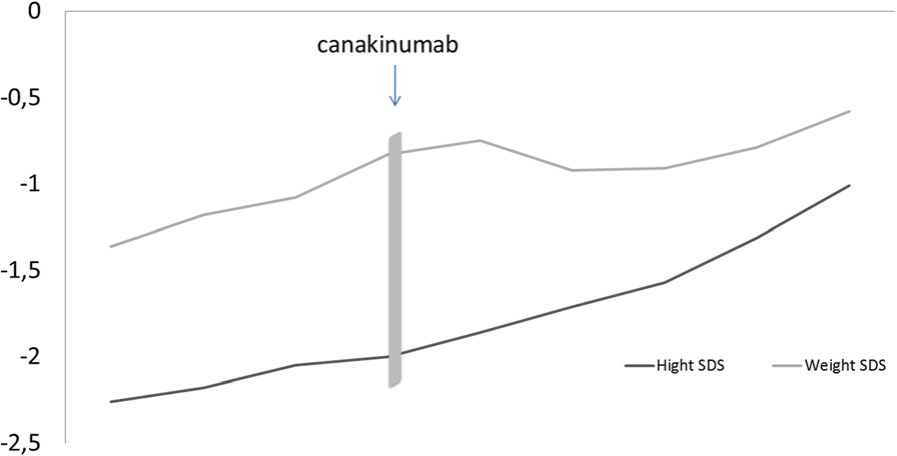
Effect of canakinumab on growth parameters (height and weight)

The laboratory findings on admission revealed an elevated CRP (184 mg/dL) and SAA (645 mg/dL) levels. Nephrotic range proteinuria was found in urine analysis (43 mg/m2/h). Because of his poor response to colchicine, severe growth retardation, and severe proteinuria due to amyloidosis, we decided to start canakinumab treatment (150 mg/month/sc) in June 2013. Informed consent about the potential side effects and the empirical aspects of the therapy was obtained. One month later, the patient was symptom-free and the inflammatory parameters almost normalized. After 26 months of follow-up, with canakinumab treatment, his complaints, inflammatory parameters (CRP; 0.03 mg/dl and SAA; 3.81 mg/dl) and proteinuria were decreased. Splenomegaly was decreased and also his growth rate returned to normal (Fig. 2), after canakinumab therapy. The mean height SDS before therapy was significantly lower than after canakinumab (−2.12 ± 0,11 vs −1.71 ± 0,14, *P* = 0,009) (Fig. 3). He was kept on 2 mg of colchicine daily. No side effects were noted.

## Discussion and evaluation

The most serious complication of FMF is the development of AA type amyloidosis which is characterized by proteinuria and is typically progressive and leads to end-stage vital organ involvement, first diagnosed by Mamou and Cattan in 1952 [15]. Renal amyloidosis has been shown to cause mortality in FMF patients [16]. In the series reported by the Turkish FMF study group, the presenting clinical features of the patients with amyloidosis secondary to FMF were as follows: 32 % proteinuria, 40 % nephrotic syndrome, and 28 % chronic renal failure [17]. The M694V mutation has been shown to be a strong risk factor of developing amyloidosis in different ethnic groups [17]. The production of the precursor to SAA is the main step in the pathogenesis of amyloidosis, which is produced by inflammatory signals, IL-1β, tumor necrosis factor (TNF)-α, and IL-6 [18]. The “gold standard” for the diagnosis of amyloidosis remains a tissue biopsy demonstrating characteristic hematoxylin and eosin changes and Congo red birefringence or metachromatic pink-violet staining with methyl violet or crystal violet [19]. The patient’s renal biopsy was evaluated with the scoring system defined for renal amyloidosis and was found as severe glomerular amyloidosis plus mild vascular and mild interstitial amyloidosis. The scoring system proposed by Sen S et al. [20] in 2010 and compared to clinical parameters by Castano et al. [21]. They have demonstrated that the severity of glomerular amyloid deposition was correlated the risk of developing end-stage renal disease and increase the risk for premature death [21]. Also they have reported proteinuria, and serum albumin and serum creatinine levels were correlated with degree of amyloidosis [21]. Also, the degree of amyloidosis was measured through parameters such as SAA protein and serum amyloid P (SAP) scintigraphy [18, 22].

Herein, we reported a FMF patient with biopsy-proven renal amyloidosis and growth retardation. He had various risk factors for amyloidosis including carrying the M694V allele, family history, and late diagnosis.

Pro-inflammatory cytokines may modulate growth patterns in children with inflammatory diseases through both systemic and local effects of the GH/IGF-1 axes [23]. It has been shown that FMF patients catch up to their growth with an effective colchicine treatment [24–26].

The aim of treatment in AA amyloidosis is the suppression, as complete as possible, of the inflammatory process responsible for the overwhelming SAA production. Colchicine is the prototype drug that decreases production of amyloidogenic precursor protein. Occasionally, colchicine inadequate patient is observed, as in our case. In such circumstances, anti IL-1 treatment options come into play. Anti- IL-1 drugs impact on amyloidosis is still unknown. Previously, there were reports of some adult cases with successful use of anti IL-1 therapy (anakinra) in renal transplant recipients [11, 12]. There are few data from pediatric patients in literature. Bilginer Y et al. [27] reported a patient who was diagnosed with FMF and Behçet’s disease and proteinuria, with normal kidney function after 18 months of anakinra treatment. Recently, Ozcakar et al. [28] showed one child patient with nephrotic syndrome in whom partial remission had been observed after 12 months of anakinra therapy.

Canakinumab is a human anti-IL-1β monoclonal antibody. Its mode of action is based on the neutralization of IL-1β signaling which may result in the suppression of the inflammation process. To the best of our knowledge, about de novo canakinumab treatment in FMF patients with AA amyloidosis is limited. Topaloglu R et al. [29] reported a patient diagnosed amyloidosis was successful treated with canakinumab.

Cetin P et al. [12] reported experience in 20 cases of adult and pediatric FMF colchicine-resistant patients who were treated with anti-IL-1 agents. Twelve patients were receiving anakinra, and eight patients were treated with canakinumab. The number of monthly and yearly attacks after IL-1 treatment significantly decreased after the biologic agent (*p* < 0.05). Hashkes P et al. [13] conducted an open-label, single-arm study in seven children with colchicine-resistant FMF. Six participants met the primary outcome with ≥50 % reduction (range 76–100 %) in the FMF attack rate. The median 28-day time-adjusted attack rate decreased from 2.7 to 0.3 (89 %). Canakinumab was shown to be effective in treating pediatric patients with colchicine-resistant FMF in this study. Another study reported that in children with colchicine-resistant FMF, monthly canakinumab 150 mg subcutaneous injections prevented FMF attacks in patients with frequent attacks, and only one of nine patients experienced an attack during the treatment period [14].

## Conclusions

Canakinumab has demonstrated a sustained clinical response in the patient affected by colchicine-resistant FMF and biopsy-proven renal amyloid deposits, blocking and significantly reducing renal damage progression. Also, we observed the normalization of the markers of inflammation inc. SAA, and the reduction of proteinuria in an overall period. Moreover, his growth pattern was improved with therapy. No adverse events, namely infectious episodes, were reported in our patient during treatment with canakinumab. We did not consider making a repeat biopsy for proteinuria completely regressed.

Our report emphasizes that the therapeutic intervention with canakinumab can treat colchicine-resistant FMF by suppressing inflammation and to prevent its most life-threatening complication, amyloidosis-related proteinuria. Further evaluations are needed in order to confirm the positive effect of canakinumab.

## Abbreviations

CKD, chronic kidney disease; CRP, C-reactive protein; FMF, Familial Mediterranean fever; IL, interleukin; SAA, serum amyloid A protein; TNF, tumor necrosis factor
